# Modeling Dual-Task Performance: Identifying Key Predictors Using Artificial Neural Networks

**DOI:** 10.3390/biomimetics10060351

**Published:** 2025-05-29

**Authors:** Arash Mohammadzadeh Gonabadi, Farahnaz Fallahtafti, Judith Heselton, Sara A. Myers, Ka-Chun Siu, Julie Blaskewicz Boron

**Affiliations:** 1 Institute for Rehabilitation Science and Engineering, Madonna Rehabilitation Hospitals, Lincoln, NE 68506, USA; 2 Department of Biomechanics and Center for Research in Human Movement Variability, University of Nebraska at Omaha, Omaha, NE 68182, USA; ffallahtafti@unomaha.edu (F.F.); samyers@unomaha.edu (S.A.M.); 3 Department of Gerontology, University of Nebraska at Omaha, Omaha, NE 68182, USA; hyeonjungkim@unomaha.edu; 4 Department of Surgery and Research Service, Nebraska-Western Iowa Veterans Affairs Medical Center, Omaha, NE 68105, USA; 5 Department of Health and Rehabilitation Sciences, University of Nebraska Medical Center, Omaha, NE 68198, USA; kcsiu@unmc.edu

**Keywords:** dual-task performance, artificial neural network (ANN), cognitive-motor integration, gait analysis, timing, speech-linguistic features, psychosocial predictors, machine learning in healthcare, cognitive assessment

## Abstract

Dual-task paradigms that combine cognitive and motor tasks offer a valuable lens for detecting subtle impairments in cognitive and physical functioning, especially in older adults. This study used artificial neural network (ANN) modeling to predict clinical, cognitive, and psychosocial outcomes from integrated gait, speech-linguistic, demographic, physiological, and psychological data collected during single- and dual-task conditions. Forty healthy adults (ages 20–84) completed physical, cognitive, and psychosocial assessments and a dual-task walking task involving cell phone use. ANN models were optimized using hyperparameter tuning and k-fold cross-validation to predict outcomes such as the Montreal Cognitive Assessment (MOCA), Trail Making Tests (TMT A and B), Activities-Specific Balance Confidence (ABC) Scale, Geriatric Depression Scale (GDS), and measures of memory, affect, and social support. The models achieved high accuracy for MOCA (100%), ABC (80%), memory function (80%), and social support satisfaction (75%). Feature importance analyses revealed key predictors such as speech-linguistic markers and sensory impairments. First-person plural pronoun used and authenticity of internal thoughts during dual-task emerged as strong predictors of MOCA and memory. Models were less accurate for complex executive tasks like TMT A and B. These findings support the potential of ANN models for the early detection of cognitive and psychosocial changes.

## 1. Introduction

Dual-task paradigms, which include walking, are increasingly recognized as a valuable framework for studying the interaction between motor and cognitive functions in real-world settings [1]. The attention demands in dual-task situations can affect cognitive and motor domains, revealing undetected declines in single-task settings [2]. These changes may be early indicators of cognitive or functional impairment [3].

Numerous studies have highlighted that individual characteristics, including age, education level, and health status, may affect multitasking ability [4]. Higher education supports cognitive reserve, helping buffer brain changes, but its effects vary. It can protect memory in healthier adults but may accelerate the decline in those at higher dementia risk [5]. This acceleration occurs because a stronger cognitive reserve can mask the early behavioral signs of dementia, delaying their detection. Therefore, when noticeable symptoms appear, the underlying disease process may have progressed significantly, leading to the perception of a more rapid decline, even though the underlying changes have been unfolding for some time. Similarly, physical fitness, emotional well-being, and lifestyle behaviors can affect how effectively individuals manage simultaneous motor and cognitive demands [6]. Understanding these complex relationships requires advanced analytical tools that account for nonlinear patterns and interactions, which traditional models like Multiple Linear Regression (MLR) may not include.

Artificial Neural Networks (ANNs) have emerged as a powerful solution for analyzing complex, nonlinear datasets in recent years [7]. Unlike conventional methods, ANNs can model intricate relationships between variables, making them particularly appropriate for studying complex systems like dual-task performance. Prior research has demonstrated the utility of ANNs in biomechanics, with applications ranging from predicting metabolic cost during movement to analyzing joint mechanics and gait dynamics [8,9,10]. Gonabadi et al. (2024) [8] demonstrated the superiority of ANNs over traditional methods in predicting metabolic costs from biomechanical data, providing a foundation for their application in other domains, including cognitive-motor integration [8].

Recent wearable sensors have made it possible to measure many features, including kinetic and kinematic movement, biomechanics, and muscle activities, during walking and under dual-task conditions. Moreover, clinical evaluations and psychological assessments provide information about people’s stress levels, personality types, and mental health elements that may affect or be related to dual-task performance. Although the amount of research on dual-tasking in older populations is increasing, it is still necessary to combine these various sources of information to identify which particular characteristics—such as speech-linguistic metrics, demographic information, health status indicators, or psychosocial metrics—are most likely to predict important clinical outcomes like mild cognitive impairment or depressive symptoms.

In line with the field of biomimetics, our study adopts an interdisciplinary approach that bridges cognitive neuroscience, biomechanics, and artificial intelligence to model complex human behaviors. Biomimetics involves the design and application of systems that mimic biological processes. Here, we simulate the integrative mechanisms of human cognitive-motor interactions under dual-task conditions by training ANNs to predict health-related outcomes from multimodal inputs. This modeling mirrors biological processing by capturing nonlinear and adaptive relationships among sensory, motor, and cognitive domains.

Furthermore, by using Artificial Intelligence (AI) tools to analyze natural speech and gait patterns—both inherently biomimetic in nature—our work enables early detection of cognitive and psychosocial decline in real-world settings. These predictive models can potentially be translated into biomimetic diagnostic tools that reflect the adaptability and complexity of human function. This aligns with biomimetics’ goal of understanding and replicating biologically inspired systems for clinical and technological innovation.

By applying ANN modeling, the present study aimed to find the optimal subset of features for predicting clinical outcomes, thereby shedding light on how dual-task cognitive performance can serve as a sensitive indicator of overall cognitive and physical health in older adults. We also implemented an MLR model using the same dataset to benchmark the predictive performance of our ANN models. This comparison highlights the added value of nonlinear modeling in capturing complex interactions among multimodal features [8,10,11,12,13]. We hypothesized that cognitive load during dual-task conditions—reflected in gait, speech-linguistic features, and internal thought patterns—can effectively predict this population’s cognitive status and psychological conditions. Including feature importance scores and Partial Dependence Plots (PDPs) further allows for interpreting how specific variables contribute to model predictions, offering insights beyond traditional statistical approaches. These tools help interpret the model’s predictions by illustrating the marginal effects of individual features, offering insights beyond standard statistical approaches. Additionally, we employed k-fold cross-validation (k = 5) to ensure robust model evaluation and enhance the reliability of our findings, reducing the risk of overfitting and improving generalizability.

## 2. Materials and Methods

### 2.1. Participants

Forty participants (22 men and 18 women) were included in the study. Their ages ranged from 20 to 84 years (50.58 ± 21.24). Participants’ average height was 170.46 ± 10.01 cm, and weight was 77.86 ± 14.60 kg, resulting in a Body Mass Index (BMI) of 26.89 ± 4.14. Individual BMI values ranged from 19.83 to 37.21. Participants were required to be healthy adults, physically active, and capable of independent ambulation, with no history of progressive neurological diseases such as multiple sclerosis or Parkinson’s disease. Exclusion criteria included the use of depression medications that could cause dizziness, cognitive impairment, or physical performance deficits, and hearing impairments (even with amplification) that affected speech perception. Additionally, individuals with an inter-aural tone threshold difference averaging over 15 decibels at 1000 Hz, those who were pregnant, or those who were breastfeeding were also excluded. Recruitment was conducted through community advertisements and university networks to ensure a diverse sample. Ethical approval was obtained from the Institutional Review Board at the University of Nebraska Medical Center (IRB #0639-19-EP). Participants provided written informed consent after being briefed about the study’s purpose, procedures, and potential risks. Participants were compensated for their time and effort.

### 2.2. Data Collection

This study utilized a range of physical, cognitive, and psychological assessments to evaluate participants’ overall functioning. Physical measures included hand dynamometry for grip strength, the Timed Up and Go test for mobility, and the Frailty Index for Elders to assess frailty risk. Cognitive assessments covered memory, processing speed, spatial orientation, and executive function, including the Montreal Cognitive Assessment, Trail Making Test, Word Fluency Test, and the Wechsler Adult Intelligence Scale (WAIS-III) Digit Symbol test. Psychological and functional measures assessed fatigue (Pittsburgh Fatigability Scale), Activities-Specific Balance Confidence Scale, Geriatric Depression Scale, Memory Functioning Questionnaire, the Perceived Stress Scale, and Pittsburgh Sleep Quality Index. These tools provide a comprehensive dataset for the relationship between individual characteristics and dual-task performance.

Data collection occurred across two laboratory visits, where participants completed both single-task and dual-task conditions to assess motor and cognitive performance. In single-task conditions, participants engaged in a three-minute conversation on an experimenter-provided mobile phone while seated. During dual-task conditions, participants walked around the perimeter of the laboratory (approximately 100 feet) at a self-selected pace for three minutes while simultaneously conversing on the same mobile phone. A low-profile, pressure-sensing walkway (3 feet wide × 12 feet long) was positioned along one side of the laboratory to capture gait data. Participants wore safety belts while walking, and a researcher walked behind them to reduce fall risk. No specific instructions were given regarding task prioritization under the dual-task condition. To ensure engagement, participants rated potential conversation topics on a 5-point Likert scale (1 = not at all interested to 5 = very interested), and one highly rated topic was selected for both task conditions. Conversation topics included personal values, travel aspirations, life challenges, responsibilities, memorable experiences, and influential figures. Participants were randomly assigned to different task conditions and conversation sets. The phone conversation, conducted with a researcher in another room, was recorded using a provided cell phone. No specific instructions were given regarding task prioritization in the dual-task condition.

### 2.3. Neural Network Inputs and Outputs

The ANN models were trained using diverse input variables encompassing multiple domains (Table 1). Demographic information included age, sex, education level, and years of occupation. Physiological data comprised body mass index, hearing and vision status, and self-reported health ratings. Lifestyle factors such as alcohol use, smoking status, and retirement status were also incorporated. Additionally, psychological measures were included, with scores derived from validated scales such as the Memory Functioning Questionnaire and the Perceived Stress Scale. Cognitive performance metrics from the dual-task conditions, including cognitive outcomes such as reaction time and task accuracy, were measured.

A software, Language Inquiry Word Count (LIWC2015) [14], was used to quantify the usage rate of differentiation words, conjunction words, prepositions, cognitive mechanisms, and words greater than six letters in the conversations. The LIWC is a computerized program that analyzes text by categorizing and quantifying language use in writing and speaking [14]. One of the categories, psychological processes, addresses emotionality, social relationships, and thinking styles related to cognitive mechanisms. Under the subscales, the thinking style category (i.e., differentiation words, conjunction words, prepositions, cognitive mechanism, and words greater than six letters) was chosen for this study to examine cognitive complexity, which refers to the richness of two components of reasoning, namely the extent to which someone differentiates between multiple competing solutions and the extent to which someone integrates among solutions. Each participant’s conversations were separately transcribed through Rev.com.

This study’s outcome measures included the ABC Scale for assessing individuals’ confidence in performing various daily activities without falling and the Montreal Cognitive Assessment (MOCA [15]) for detecting mild cognitive impairment. The Trail Making Test [16], comprising Parts A (TMTA) and B (TMTB), was administered to gauge visual attention, task-switching ability, and executive functioning [16]. The Memory Functioning Questionnaire (MFQ [17]) captured self-reported memory performance, and affective states were evaluated using the Positive and Negative Affect Schedule (PANAS [18]), with separate subscales for Positive Affect (PA) and Negative Affect (NA). Perceived stress over the past month was measured via the Perceived Stress Scale (PSS [19]). Additionally, anxiety was analyzed using the State-Trait Anxiety Inventory (STAXI [20]), which distinguishes between temporary (state) and chronic (trait) anxiety, as well as overall anxiety expression. Core personality traits—extraversion, agreeableness, conscientiousness, neuroticism, and openness—were assessed through the Big Five Inventory (BFI [21,22]). At the same time, sleep quality and disturbances were evaluated using the Pittsburgh Sleep Quality Index (PSQI [23]). The Geriatric Depression Scale (GDS [24]) measured depressive symptoms specifically in older adults. Lastly, social support was quantified using the Social Support Rating Scale (SSRS [25]), capturing both the size of one’s network (Social Support [N]) and satisfaction with that network (Social Support [S]).

In this study, we implemented a single ANN model configured to simultaneously predict multiple heterogeneous outcomes (e.g., MOCA, GDS, ABC, speech-linguistic, and memory scores) based on the same set of shared input features. This multi-output structure allows the model to learn shared patterns across cognitive, psychological, and physical domains, improving predictive coherence and efficiency. This approach follows previously published frameworks using unified neural networks for multi-target prediction in interrelated clinical domains [8,10,11,12,13].

### 2.4. Artificial Neural Network Modeling

The ANN models were designed to predict clinical outcomes using a comprehensive set of input features, including demographic, physiological, gait, and speech-linguistic variables. Each model was structured with three main layers: (1) an input layer corresponding to the number of predictor variables; (2) a single hidden layer, whose size was optimized through systematic grid and random search; and (3) an output layer representing each target outcome. The hidden layer used a log-sigmoid activation function (logsig) to capture nonlinear interactions, while the output layer used a purelin (linear) activation function to support continuous prediction [8,10,11,12]. The models were trained using the Levenberg–Marquardt backpropagation algorithm, chosen for its efficiency in fitting nonlinear mappings within relatively small datasets. A 5-fold cross-validation strategy (k = 5) was applied to evaluate model robustness and avoid overfitting [8]. During each fold, one subset of the data was used for testing, while the remaining four were used for training, and this process was repeated until each subset had been used for testing once. We computed Root Mean Squared Error (RMSE) and R-squared (R^2^) values across all folds to assess model reliability and performance. Additionally, feature importance scores were extracted to determine the relative contribution of each predictor to the ANN’s output. PDPs were generated to visualize the marginal effects of influential features on the predicted outcomes and enhance interpretability.

The architecture and training configuration of the ANN models are summarized in Table 1. This includes the number of input features, the number of neurons in the hidden layer, the output structure, activation functions used in each layer, the learning algorithm (Levenberg–Marquardt), and the evaluation method (5-fold cross-validation).

**Figure 1:**
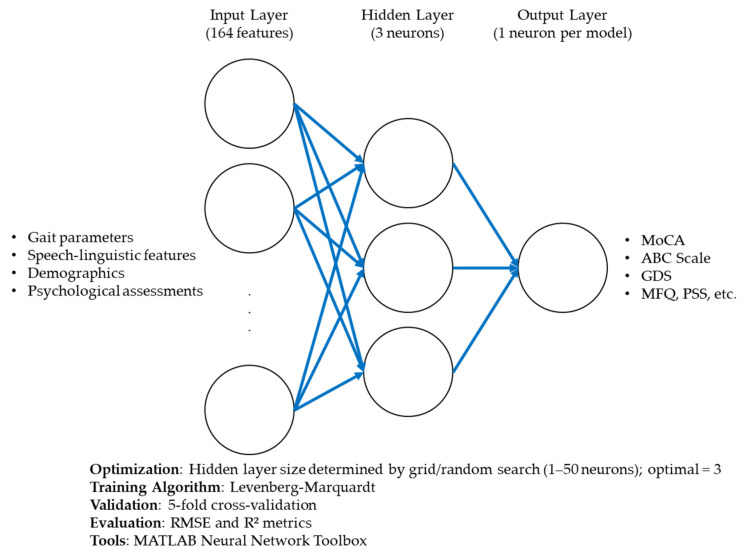
Schematic representation of the artificial neural network (ANN) architecture used for outcome prediction. The model includes an input layer composed of multi-domain features (e.g., gait, speech-linguistic, psychological, sensory, and demographic inputs), a single hidden layer with an optimized number of neurons determined via hyperparameter tuning, and an output layer corresponding to a single clinical or psychosocial outcome. The ANN was trained using the Levenberg–Marquardt backpropagation algorithm with 5-fold cross-validation. Model performance was assessed using root mean squared error (RMSE) and R-squared values (R^2^), and interpretability was enhanced with feature importance scores and partial dependence plots (PDPs). The blue lines represent weighted connections between neurons across layers, indicating information flow during network training and prediction.

Figure 1 illustrates the detailed architecture of the ANN used in this study, including the input layer, optimized hidden layer, and output unit. It also outlines the training parameters and performance evaluation strategy.

### 2.5. Artificial Neural Network Architecture and Optimization

The ANN implemented in this study was designed to model the complex relationships between input features and outcomes. The network architecture included a single hidden layer with three neurons, as specified in Table 2. The ANN utilized all input variables simultaneously, including demographic, physiological, psychological, and lifestyle features, to account for their interactions in predicting outcomes.

The dataset was split into training (70%), validation (15%), and testing (15%) subsets to ensure robust model development. Specifically, out of 40 participants, 28 were allocated to the training set, 6 to the validation set, and 6 to the test set in each fold of the 5-fold cross-validation procedure. A Levenberg–Marquardt backpropagation algorithm was employed for training, as it is well-suited for solving nonlinear problems and offers efficient convergence for smaller datasets [8,10,11,12,13]. A subject-wise data split was used to avoid overfitting and enhance generalizability, ensuring that data from each subject was exclusive to one subset.

Given the small sample size, we opted for ANNs rather than deep learning methods to ensure the model’s suitability for our dataset. To enhance the reliability of our ANN models and strike a balance between convergence speed and stability within the smaller dataset [26], we employed a robust optimization approach inspired by methodologies from Nogueira et al. [26] and Gonabadi et al. [8,10]. This process included systematic hyperparameter tuning using grid and random search techniques to identify the optimal hidden layer sizes for each ANN model [26]. The optimization strategy leveraged performance metrics such as Mean Squared Error (MSE) and R-squared (R) values to evaluate and compare configurations across cross-validation folds. This systematic process utilized nested for-loops in MATLAB R2024b (MathWorks, Natick, MA, USA), varying hidden layer sizes (from 1 to 50 neurons), and iterating each parameter combination 1000 times to ensure stability and minimize stochastic effects. Each configuration was assessed based on MSE values for training, validation, test sets, and correlation coefficients (R-values) to determine predictive accuracy. We observed that increasing the number of neurons in a single hidden layer initially enhanced model performance, reflected by lower MSE and higher R-values, signifying improved predictive accuracy. However, beyond a certain threshold of complexity, the model exhibited diminishing returns and occasional overfitting, as indicated by higher MSE values on the validation set. This analysis allowed us to pinpoint an optimal architecture that balanced complexity with predictive reliability. These efforts were further informed by prior optimization strategies described in Gonabadi et al. [8,10], ensuring our approach integrated best practices for ANN model development. We developed ANN models for high performance and reliability to ensure an optimal balance between predictive accuracy and computational efficiency.

In summary, we used a single-hidden-layer feedforward ANN structure due to its effectiveness in capturing nonlinear relationships in small datasets [8,10,11,12,13]. We implemented grid and random search strategies across 1 to 50 neurons to determine the optimal number of neurons in the hidden layer. We selected the final configuration based on the combination that minimized RMSE and maximized R-values across 5-fold cross-validation. The 70% training, 15% validation, and 15% test data split was adopted based on standard practice, ensuring sufficient capacity for training while maintaining robustness during validation and testing [8,10,11,12,13].

The modeling process was done using MATLAB’s Neural Network Toolbox, supplemented by custom MATLAB scripts designed for this research. The ANN model’s performance was assessed using regression analysis, comparing predicted and actual outcomes across all phases of the dataset. Metrics, including MSE and R-values, were used to evaluate predictive accuracy, while residual analysis provided insights into error distributions. These methodological choices ensured the ANN’s capacity to effectively model the intricate, nonlinear interactions among input features.

### 2.6. Statistical Analyses

To evaluate the performance of the ANN model, standard classification metrics, including Accuracy, Precision, Recall, and *F*1 Score, were calculated using the confusion matrix components: **(A) True Positives (TP)** are cases where the model correctly predicts a positive outcome. **(B) True Negatives (TN)** are cases where the model predicts a negative outcome correctly. **(C) False Positives (FP)** are cases where the model incorrectly predicts a positive outcome. **(D) False Negatives (FN)** are cases where the model incorrectly predicts a negative outcome. These metrics are computed under an acceptable error margin of ±10%, ensuring robustness in prediction evaluation. This threshold was selected based on prior literature in neural network modeling applied to biomedical and behavioral data, where prediction errors within ±10% are often regarded as clinically or functionally acceptable [8,10,11,12,13]. This approach provides a more interpretable measure of the model’s performance within a realistic margin of variability, mainly when predicting continuous clinical outcomes. The metrics that we used are *Accuracy* [27], *Precision*, *Recall* (Sensitivity), and *F*1 Score [28], Equations (1)–(4).

*Accuracy* measures the overall correctness of the model by quantifying the ratio of correctly predicted outcomes to the total number of outcomes.

(1) $$Accuracy=\frac{TP+TN}{TP+TN+FP+FN}$$

*Precision* evaluates the model’s ability to identify positive outcomes and minimize false positives correctly.

(2) $$Precision=\frac{TP}{TP+FP}$$

*Recall* quantifies the model’s ability to identify positive cases and reduce false negatives.

(3) $$Recall=\frac{TP}{TP+FN}$$

The *F*1 Score provides a harmonic mean of precision and recall, offering a balanced measure, especially for imbalanced datasets.

(4) $$F1\;Score=2\times \frac{Precision\times Recall\;}{Precision+Recall}$$

In this study, prediction accuracy for each outcome was calculated based on whether the predicted value fell within ±10% of the actual (observed) value. This threshold was chosen based on prior literature utilizing similar ANN models for clinical and behavioral predictions [8,10,11,12,13]. For example, a predicted score was considered accurate if it deviated by no more than ±10% of the actual score. This operational definition provided a practical and interpretable metric for assessing the model’s performance across diverse clinical and psychosocial outcomes.

In line with previous studies [8,10,11,12,13], to establish a baseline for model performance, we additionally implemented an MLR model. The MLR was applied to the same dataset as the ANN, using identical outcome variables and input features. The MLR served as a linear reference point to compare the predictive capabilities of the ANN in modeling complex and nonlinear interactions. Evaluation metrics, including RMSE, accuracy, recall, precision, and *F*1-score, were computed for both models across training, validation, and test partitions. This comparative framework allowed us to assess the relative benefits of using ANN over traditional regression methods in multimodal data contexts.

## 3. Results

### 3.1. Overall

The ANN demonstrated robust predictive performance for all dual-task outcomes. Optimized with a single hidden layer containing three neurons, the model’s architecture effectively captured the nonlinear relationships between input features and the predicted outcomes (Table S1). Table S1 lists all input features used in the predictive models, along with their associated abbreviations and detailed descriptions, serving as a reference for interpreting feature importance across outcomes. This configuration was validated during the training and testing, ensuring its generalizability and reliability. The network achieved consistent performance metrics across the validation and testing datasets, highlighting its capacity to model complex interactions within the data.

**Figure 2:**
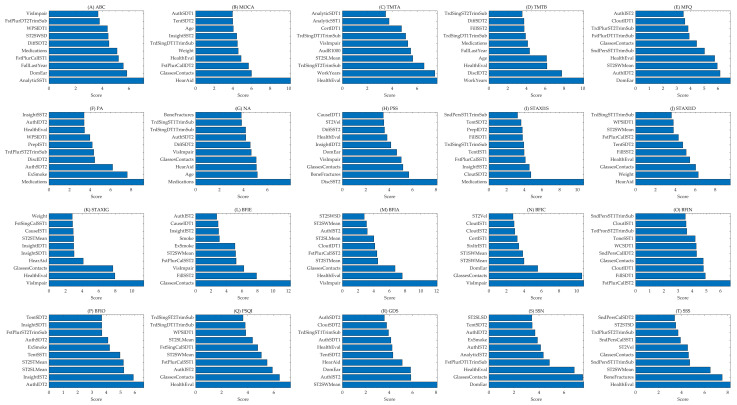
This figure provides an overall view of the relationship between each outcome and the corresponding features (the top ten important features). It serves as a comprehensive summary illustrating how various features impact the outcomes. Each plot within the figure represents a probability density function (PDF), offering insight into the strength and distribution of feature importance across the outcomes. This visualization emphasizes the key contributions of specific features in predicting each outcome, highlighting the interdependence between features and their respective outcomes in a concise overview. The complete names of features and outputs are described in Table S1.

Table 2 also represents the architecture of the ANN used in this study and presents the accuracy levels achieved for each outcome. The table provides detailed information on the specific features the ANN model utilizes, along with their corresponding accuracy levels. Notably, this study employs a single ANN model to predict multiple outcomes simultaneously, leveraging a shared network architecture to optimize predictive performance across all outcomes. Each column represents an outcome, while the rows depict the performance metrics, including accuracy and key architecture parameters. The consistency of using a single ANN for all outcomes reflects the network’s ability to generalize across diverse prediction tasks. This unified approach not only simplifies the model structure but also highlights the versatility and robustness of the ANN in handling complex, multifaceted datasets. The results demonstrate the effectiveness of this architecture in achieving reliable predictions across a wide range of outcomes. The top features predicting each outcome are shown in Figure 2, while their abbreviations and descriptions appear in Table S1.

### 3.2. Model Training and Overview

The dataset was split into training (70%), validation (15%), and test (15%) subsets, and a feedforward ANN with three hidden neurons was used to predict multiple outcomes simultaneously. Model performance was assessed via root mean squared error (RMSE) for continuous outcomes and by accuracy, recall, precision, and *F*1 score for the classification task.

### 3.3. Variation in Predictive Success by Outcome

Table 2 summarizes the performance metrics for each outcome. Training RMSE: Ranged from 1.44 (for MOCA) to 10.75 (for TMT B), reflecting variability in how well the model fits different continuous outcomes. Validation and Test RMSE: Most measures showed slightly higher RMSE on the validation and test sets than on the training set, indicative of some generalization error. Notably, MOCA maintained a relatively low RMSE (1.77 validation; 1.50 test), suggesting that the model learned this measure more robustly. Accuracy (Classification): MOCA, a measure of task-switching ability, reached 100% accuracy on the test set—a standout result. Although the model achieved 100% accuracy for MOCA within the ±10% threshold, this should be interpreted cautiously. This reflects the model consistently producing values within an acceptable clinical margin rather than a perfect score-level prediction. Instead, it suggests the model could consistently estimate values within a clinically reasonable margin of error. This interpretation aligns with standard practices in evaluating regression-based ANN outputs in clinical prediction tasks [8,10,11,12,13]. Other relatively high accuracies included ABC (80%), MFQ (80%), and Social Support Satisfaction (75%). In contrast, TMT A achieved only 17.5% accuracy, indicating an incredible difficulty in predicting this more complex executive function measure. *F*1 Scores: Aligned closely with accuracy for most outcomes, with MOCA scoring the highest (*F*1 = 1.00). MFQ and ABC achieved an *F*1 score of ~0.80, while Social Support Satisfaction reached 0.74.

In addition to developing and evaluating the ANN model, we implemented an MLR model to serve as a baseline for performance comparison. Both models were applied to the same dataset, and their predictive performances were assessed using standard metrics including root mean square error (RMSE), accuracy, recall, precision, and *F*1-score. As shown in Table 2, the ANN model consistently outperformed MLR across all outcome variables. Specifically, the ANN achieved a lower RMSE (8.52 vs. 11.22) and higher classification metrics (accuracy: 40.5% vs. 32.4%; recall: 40.1% vs. 32.6%; precision: 40.5% vs. 32.4%; *F*1-score: 0.40 vs. 0.33). These results highlight the ANN’s superior capability in modeling complex, nonlinear relationships within the multimodal dataset.

The RMSE for each outcome was calculated by first computing the RMSE in the five k-fold (k = 5) cross-validation folds. These individual RMSE values were then averaged to obtain the aggregated RMSE reported in Table 2. This method ensures a stable estimation of model performance and reduces the bias that could arise from a single train-test split [8,10,11,12,13].

**Figure 3:**
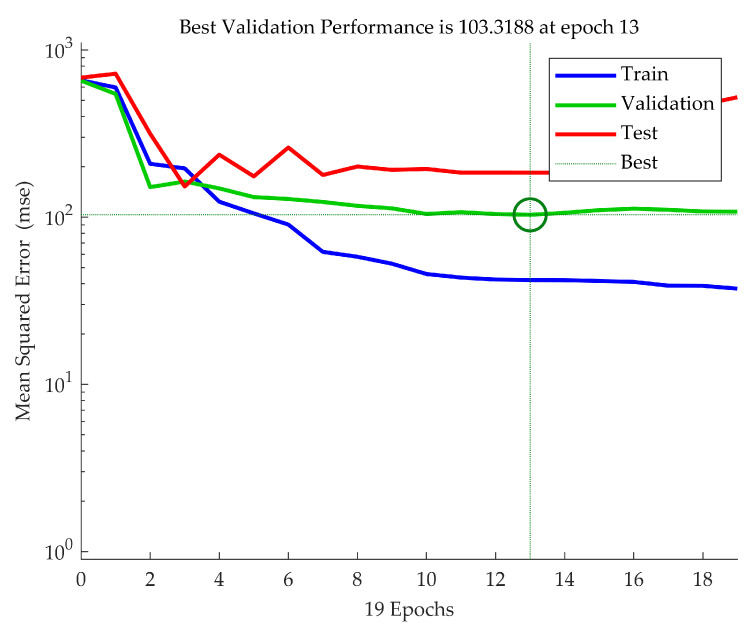
**Mean Squared Error (MSE) performance plot of the ANN model across 19 training epochs.** The blue, green, and red curves represent training, validation, and test errors. The best validation performance was achieved at epoch 13 with an MSE of 103.32, demonstrating convergence and stability across dataset partitions. The green circle indicates the point of best validation performance, achieved at epoch 13, where the MSE was minimized for the validation dataset.

To visualize the training process and model stability, we plotted the MSE for the training, validation, and test sets across epochs (Figure 3). Figure 3 indicates that the model achieved its best validation performance at epoch 13 (MSE ≈ 103.3). The training curve showed consistent downward trends, while the validation and test errors remained stable after early epochs, suggesting effective generalization without significant overfitting. These results reinforce the reliability of the ANN models under our cross-validation scheme.

**Figure 4:**
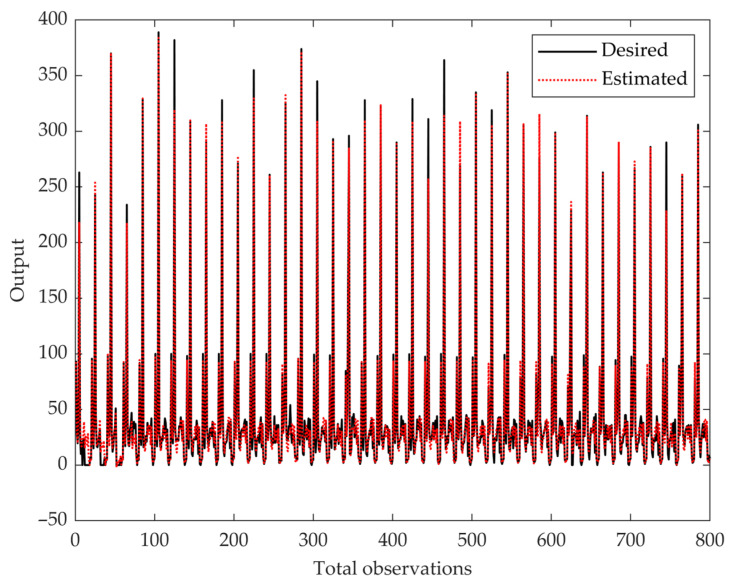
**Comparison of the desired (black line) and ANN-estimated (red dotted line) outputs across total observations.** The close alignment between the predicted and actual values demonstrates strong agreement and highlights the model’s prediction accuracy.

In addition to the MSE performance curve, we visualized the ANN’s predictive accuracy by plotting all samples’ estimated and desired outputs (Figure 4). The red dotted line represents the estimated outputs, while the black line represents the target values. The close alignment between these two lines indicates the model’s ability to learn and generalize patterns across the dataset, demonstrating reliable predictive performance across various outcome levels.

### 3.4. Overall Performance Across Outcomes

The following are the high-performing measures.

***MOCA:*** As a broad cognitive screener assessing multiple domains, such as visuospatial abilities, memory, executive function, and attention, the Montreal Cognitive Assessment still achieved perfect classification metrics (accuracy/recall/precision/*F*1 all ~100%). This suggests that, given the features used, the model could readily distinguish participants above or below the specified MOCA threshold.

***ABC:*** With 80% accuracy and an *F*1 of 0.79, the model reliably distinguished participants above or below an impairment threshold, suggesting that the cognitive dual-task performance could capture a balance score.

***MFQ:*** With 80% accuracy and an *F*1 of 0.8, the model reliably distinguished participants above or below an impairment threshold, suggesting that the cognitive features (e.g., gait/speech parameters) capture relevant perceived memory abilities, frequency of forgetfulness, and confidence in everyday memory tasks.

*Positive Affect and Social Support Satisfaction:* Both also reached high classification performance (*F*1 ≥ 0.74), implying that psychosocial states can be effectively predicted from the combined demographic, gait, and speech-linguistic features.

The following are the challenging measures.

***TMT A:*** Despite being related to executive functioning, it showed notably lower accuracy (17.5%) and *F*1 (0.17), implying greater complexity or insufficiently predictive features for this outcome.

***Other Personality and Mood Scores:*** Measures like GDS (accuracy = 17.5%, *F*1 = 0.18) and Neuroticism (25% accuracy, *F*1 = 0.25) were also less accurately predicted, hinting at potential variability in how these constructs manifest across individuals or the need for more tailored features.

### 3.5. Interpretation of RMSE for Continuous Outcomes

When considered as continuous scores (reflected by RMSE), specific measures demonstrated moderate fit even if their classification performance was low. For instance, TMT A’s test RMSE (7.82) was lower than its training RMSE (8.17), suggesting that while the classification threshold was not well captured, the model still reduced overall error for raw TMT A scores. This dual perspective (RMSE vs. accuracy) determined the importance of looking at continuous and categorical metrics in evaluating model performance. Overall, the ANN performed best at predicting ABC, MOCA, MFQ, and Social Support Satisfaction, with consistently higher accuracy, precision, recall, and *F*1 scores. This indicates that simple attention/switching measures and specific psychosocial attributes (e.g., affect, satisfaction with social support) were easier to capture using the available demographic, dual-task cognitive, and gait features. In contrast, more complex executive-function tasks (TMT A) and certain mood or personality scales (e.g., GDS, Neuroticism) proved more challenging for the model, possibly requiring additional or more nuanced features to improve predictive power.

## 4. Discussion

The present findings showed a strong predictive capability of the ANN for select psychological and cognitive outcomes when incorporating dual-task features alongside demographic variables. These results highlight how task interference and cognitive load, which arise naturally when individuals perform simultaneous walking and cognitive activities, can be closely linked with performance on standardized cognitive assessments. In this context, the dual-task parameters (e.g., alterations in gait and speech-linguistic performance under cognitive load situations) may serve as windows into one’s cognitive reserve. This emphasizes the potential clinical importance of assessing the interaction between motor and cognitive processes, which are crucial for maintaining functional independence and identifying early signs of cognitive decline.

The comparative analysis showed that the ANN substantially outperformed the MLR model across all key metrics, including RMSE, accuracy, and *F*1 score. This finding reinforces the importance of using machine learning approaches like ANN to model complex, nonlinear relationships in dual-task cognitive-motor assessments. Figure 3 visually confirms the model’s training stability, highlighting minimal overfitting and successful early convergence. Additionally, Figure 4 demonstrates the ANN model’s ability to closely align predicted and actual outcomes across observations, reinforcing its robustness and generalizability.

The ANN model achieved 100% accuracy and a perfect *F*1 score (1.00) for MOCA and the MFQ. These results suggest that demographic, health, gait, and speech-linguistic features effectively capture markers related to general cognitive functioning (as reflected by MOCA) and self-reported memory functioning (as gauged by MFQ). MOCA provides a comprehensive evaluation of multiple cognitive domains. It assesses attention, memory, executive function, language, and visuospatial abilities [29]. This multidimensional approach allows the MOCA to detect mild impairments and identify subtle cognitive deficits that narrower assessments might overlook. Furthermore, recent findings indicate that individuals with hearing or vision impairments tend to score lower on the MOCA than those with intact sensory acuity, even after accounting for these deficits [30]. This observation aligns with our model’s demonstration that medical factors, such as the use of hearing aids, can meaningfully influence MOCA score predictions.

Past reviews indicate that subjective memory complaints often reflect an interplay between genuine cognitive decline and emotional factors such as depressive symptoms [31,32]. In our study, the authenticity of internal thoughts during dual-task conditions was the strongest predictor of MFQ scores. This finding suggests that how individuals self-reflect, particularly under cognitive load, can reveal significant insights into subjective memory functioning. Higher authenticity might amplify awareness of subtle memory lapses, paralleling observations that contingent self-esteem increases vulnerability when performance standards are threatened [33]. In other words, when people remain true to their emotional states during challenging tasks, they may report more issues or align their self-assessments more closely with actual memory performance. These results emphasize the value of considering objective tests and how individuals perceive and narrate their cognitive experiences.

The Activities-Specific Balance Confidence (ABC) Scale measures an individual’s confidence in maintaining balance during various daily activities and was best predicted by analytic scores calculated for speech in single-task 1. This finding is intriguing, as it suggests that how participants structured their speech, even in a low-demand, single-task context, may reflect underlying cognitive processes linked to balance confidence. It aligns with research showing that cognitive function, particularly executive control and attentional resources, is tightly linked to physical activity and mobility outcomes in older adults. For example, Kumar et al. (2022) demonstrated that older adults in India who engaged in regular physical activity had significantly better cognitive functioning than their less active peers, highlighting how maintaining physical activity benefits cognitive and functional domains [34]. Given that balance confidence influences mobility and fall risk, our findings suggest that cognitive markers like analytic speech could reflect broader functional abilities, including physical confidence.

The speech-linguistic features extracted through LIWC analysis emerged as strong predictors for multiple outcomes. For instance, the frequency of first-person plural pronouns (e.g., “we”, “us”) was the top predictor of MOCA scores, suggesting a link between inclusive language use and cognitive performance [14]. This aligns with prior studies indicating that social and self-referential language markers are associated with aging populations’ cognitive engagement and executive functioning [14]. Additionally, markers of authenticity, cognitive processing, and tone captured from speech samples during dual-task walking were influential in predicting memory and affect-related outcomes [14]. These findings highlight the sensitivity of naturalistic language to reflect underlying cognitive and emotional states, mainly when individuals are engaged in cognitively demanding conditions such as dual-task walking [14]. This underscores the value of integrating speech-linguistic behavior into AI-based predictive models for clinical screening.

One limitation of this study is the relatively small sample size of older adults. This led us to include participants across a wide age range rather than focusing solely on older individuals. As a result, the findings may not be fully representative or specific to the aging population. Future research will address this limitation by expanding the dataset to include a larger sample of older adults, allowing the model to capture age-specific patterns and improve generalizability to older populations more accurately. With a relatively small dataset, these models are at risk of overfitting, which can affect the reliability of their predictions. Additionally, ANNs are often seen as “black-box” models, making it difficult to interpret how specific inputs contribute to the outputs, even with techniques like feature importance scoring. Although we employed k-fold cross-validation to minimize overfitting and evaluate the model’s generalizability, the small sample size remains a key limitation. As the reviewer noted, a limited dataset may constrain the model’s ability to generalize across broader populations, and future research should involve larger, more diverse cohorts to validate and extend our findings. Another limitation is the sample size. While informative, this study included 40 participants, which may not fully represent the diversity of dual-task performance across different populations. Expanding the sample size in future studies would improve the model’s generalizability and robustness. While the ANN models demonstrated strong predictive accuracy for several outcomes (e.g., MOCA, ABC, MFQ), the prediction performance for some variables, such as Trail Making Test A (TMT A) and Trail Making Test B (TMT B), was notably lower. This reduced accuracy likely reflects the complex nature of these executive functioning tasks, which may depend on cognitive processes not fully represented by the input features used in this study. The limited sample size may have also restricted the model’s ability to generalize to these specific outcomes. These findings suggest that future work should incorporate more targeted neurocognitive features or expanded datasets to improve predictive performance for higher-order cognitive tasks. Furthermore, while this study employed a specific ANN model, exploring other architectures or AI approaches, such as ensemble learning methods, convolutional neural networks, or decision tree-based models like gradient boosting, may yield improved performance and interpretability. Another potential limitation is that participants were not given explicit instructions regarding task prioritization during dual-task conditions. This may have introduced variability in performance, as individuals could have unconsciously prioritized either the cognitive (speech) or motor (gait) component. While this design choice aimed to mimic naturalistic conditions, it may affect interpretability and model generalizability. Future studies should consider standardizing or systematically manipulating task priority to better control for this variability. Incorporating a comparative analysis of these methods could provide further insights into the strengths and limitations of various AI techniques in this domain. Addressing these limitations in future work could involve increasing the sample size, using external validation datasets, and applying advanced methods to improve the transparency and interpretability of ANN models.

## 5. Conclusions

This study’s findings reveal the strengths and limitations of employing a single ANN model to predict diverse psychological, cognitive, and subjective physical outcomes. This suggests that future improvements could include expanding the feature set to capture fine-grained executive functions, such as incorporating sensor-based motor measures or higher-resolution speech analytics, and testing more profound or recurrent neural network architectures better suited to complex temporal patterns. Moreover, adopting longitudinal study designs could clarify whether these multi-domain indicators predict baseline performance and serve as early cognitive or emotional decline markers. It is also worth noting that mild cognitive impairment (MCI) does not continually develop into full-blown dementia, just like someone with depression may not remain depressed indefinitely. Recognizing these nuances in early predictive models can help people receive interventions or support sooner, potentially slowing or preventing more serious declines in their cognitive or emotional well-being. Ultimately, while this study demonstrates the potential of ANN models in clinical prediction, it also highlights the need for tailored approaches when modeling more intricate cognitive processes or multifactorial mental health outcomes.

## Figures and Tables

**Table 1:** Architecture and configuration parameters of the Artificial Neural Network (ANN) models used to predict clinical, cognitive, and psychosocial outcomes. Each model was trained independently using the Levenberg–Marquardt backpropagation algorithm and evaluated with 5-fold cross-validation.

Parameter	Description
Number of Input Features	132 (speech-linguistic, gait, physiological, and demographic features)
Number of Hidden Layers	1
Hidden Layer Size	3 neurons (optimized via hyperparameter tuning)
Output Layer	1 node per model (continuous clinical/cognitive outcome)
Activation Function (Hidden)	Tangent sigmoid
Activation Function (Output)	Linear
Training Algorithm	Levenberg–Marquardt backpropagation
Cross-Validation	5-fold
Evaluation Metrics	Root Mean Squared Error (MSE), R-squared (R^2^), Feature Importance Scores, PDPs

**Table 2:** Comparative analysis of Artificial Neural Network (ANN) and Multiple Linear Regression (MLR) performance metrics across different outcome models. The table summarizes key ANN model parameters, including the ratios for training, validation, and testing (unseen data), hidden layer configurations, and input delays. It also reports predictive performance indicators such as RMSE, accuracy (within a ±10% error margin), recall, precision, and *F*1 score across training, validation, testing, and total datasets. Additionally, we included the performance of the MLR model as a baseline comparison to evaluate the added value of the ANN approach. The ANN outperformed MLR across all evaluation metrics, supporting its utility in modeling complex, multimodal features and nonlinear interactions. This comparative analysis highlights the ANN’s superior predictive accuracy and correlation coefficients while acknowledging the baseline capacity of the MLR.

	All Outputs	(A) ABC	(B) MOCA	(C) TMTA	(D) TMTB	(E) MFQ	(F) PA	(G) NA	(H) PSS	(I) STAXI1S	(J) STAXI1D	(K) STAXIG	(L) BFIE	(M) BFIA	(N) BFIC	(O) BFIN	(P) BFIO	(Q) PSQI	(R) GDS	(S) SSN	(T) SSS
ANN:																					
Training Ratio (%)	70.00																				
Validation Ratio (%)	15.00																				
Test Ratio (%)	15.00																				
Hidden Layer Size (neurons)	3.00																				
Training Performance (rmse)	6.49	7.93	1.44	8.17	10.75	10.11	7.70	5.03	4.78	5.38	4.99	7.85	5.49	7.51	6.88	7.60	7.91	2.79	1.86	1.79	0.67
Validation Performance (rmse)	10.16	6.70	1.77	15.02	19.48	32.60	7.00	7.60	5.97	3.44	5.58	4.27	3.64	2.90	6.71	4.76	6.22	3.44	1.56	2.09	0.37
Test Performance (rmse)	13.58	5.06	1.50	7.82	20.40	47.02	6.07	5.55	7.21	9.66	3.09	8.19	7.66	13.10	12.90	8.81	13.29	5.49	0.44	1.68	0.29
All data Performance (rmse)	8.52	7.39	1.50	9.47	14.15	23.72	7.37	5.57	5.40	6.00	4.85	7.48	5.65	8.15	8.05	7.45	8.72	3.43	1.68	1.82	0.59
*Accuracy* (%)	40.50	80.00	100.00	17.50	47.50	80.00	32.50	15.00	12.50	52.50	32.50	35.00	37.50	42.50	42.50	25.00	40.00	7.50	17.50	17.50	75.00
*Recall* (%)	40.10	78.05	100.00	17.07	47.50	80.00	32.50	15.00	12.50	51.22	33.33	35.00	38.46	39.53	41.46	25.00	38.10	7.50	17.50	17.50	73.17
*Precision* (%)	40.50	80.00	100.00	17.50	47.50	80.00	32.50	15.00	12.50	52.50	32.50	35.00	37.50	42.50	42.50	25.00	40.00	7.50	17.50	17.50	75.00
*F*1 Score	0.40	0.79	1.00	0.17	0.48	0.80	0.33	0.15	0.13	0.52	0.33	0.35	0.38	0.41	0.42	0.25	0.39	0.08	0.18	0.18	0.74
**MLR:**																					
All data Performance (rmse)	11.22	8.13	2.42	14.31	32.81	35.33	9.86	8.15	6.57	13.61	6.32	7.55	12.39	18.67	16.29	8.53	11.32	6.29	1.87	3.35	0.62
*Accuracy* (%)	32.38	70.00	82.50	10.00	17.50	62.50	35.00	17.50	22.50	30.00	35.00	37.50	15.00	32.50	32.50	22.50	30.00	17.50	2.50	20.00	55.00
*Recall* (%)	32.64	71.79	82.50	10.00	17.50	64.10	35.90	17.50	22.50	30.00	34.15	37.50	15.00	33.33	32.50	22.50	31.58	17.50	2.50	19.51	55.00
*Precision* (%)	32.38	70.00	82.50	10.00	17.50	62.50	35.00	17.50	22.50	30.00	35.00	37.50	15.00	32.50	32.50	22.50	30.00	17.50	2.50	20.00	55.00
*F*1 Score	0.33	0.71	0.83	0.10	0.18	0.63	0.35	0.18	0.23	0.30	0.35	0.38	0.15	0.33	0.33	0.23	0.31	0.18	0.03	0.20	0.55

## Data Availability

The raw data supporting the conclusions of this article will be available from the authors on request.

## Supplementary Materials

The following supporting information can be downloaded at: https://www.mdpi.com/article/10.3390/biomimetics10060351/s1, Table S1: The ANN robust predictive performance for all dual-task outcomes.

## References

[1] Langeard A., Torre M.M., Temprado J.J. (2021). A Dual-Task Paradigm Using the Oral Trail Making Test While Walking to Study Cognitive-Motor Interactions in Older Adults. Front. Aging Neurosci..

[2] Sleimen-Malkoun R., Temprado J.-J., Berton E. (2013). Age-related dedifferentiation of cognitive and motor slowing: Insight from the comparison of Hick-Hyman and Fitts’ laws. Front. Aging Neurosci..

[3] Beauchet O., Annweiler C., Dubost V., Allali G., Kressig R.W., Bridenbaugh S., Berrut G., Assal F., Herrmann F.R. (2009). Stops walking when talking: A predictor of falls in older adults?. Eur. J. Neurol..

[4] Fernández-Lago H., Bosch-Barceló P., Sánchez-Molina J.A., Ambrus M., Rio D., Fernández-Del-Olmo M.Á. (2024). Cognitive reserve and executive functions in dual task gait performance in Parkinson’s disease. Exp. Brain Res..

[5] Zahodne L.B., Mayeda E.R., Hohman T.J., Fletcher E., Racine A.M., Gavett B., Manly J.J., Schupf N., Mayeux R., Brickman A.M. (2019). The role of education in a vascular pathway to episodic memory: Brain maintenance or cognitive reserve?. Neurobiol. Aging.

[6] Mandolesi L., Polverino A., Montuori S., Foti F., Ferraioli G., Sorrentino P., Sorrentino G. (2018). Effects of Physical Exercise on Cognitive Functioning and Wellbeing: Biological and Psychological Benefits. Front. Psychol..

[7] Valenzuela O., Catala A., Anguita D., Rojas I. (2023). New Advances in Artificial Neural Networks and Machine Learning Techniques. Neural Process. Lett..

[8] Mohammadzadeh Gonabadi A., Fallahtafti F., Antonellis P., Pipinos I.I., Myers S.A. (2024). Ground Reaction Forces and Joint Moments Predict Metabolic Cost in Physical Performance: Harnessing the Power of Artificial Neural Networks. Appl. Sci..

[9] Zhang L., Li Z., Hu Y., Smith C., Farewik E.M.G., Wang R. (2021). Ankle Joint Torque Estimation Using an EMG-Driven Neuromusculoskeletal Model and an Artificial Neural Network Model. IEEE Trans. Autom. Sci. Eng..

[10] Mohammadzadeh Gonabadi A., Fallahtafti F., Pipinos I.I., Myers S.A. (2025). Predicting lower body joint moments and electromyography signals using ground reaction forces during walking and running: An artificial neural network approach. Gait Posture.

[11] Narayan J., Dwivedy S.K. (2021). Biomechanical Study and Prediction of Lower Extremity Joint Movements Using Bayesian Regularization-Based Backpropagation Neural Network. J. Comput. Inf. Sci. Eng..

[12] Mohammadzadeh Gonabadi A., Fallahtafti F., Burnfield J.M. (2024). How Gait Nonlinearities in Individuals Without Known Pathology Describe Metabolic Cost During Walking Using Artificial Neural Network and Multiple Linear Regression. Appl. Sci..

[13] Gonabadi A.M., Connelly E., Williams N., Massey J., Solsrud A., Janovec D., Chaidez V., Scofield D., Burnfield J. (2025). Prediction of Overall Sleep Score Outcomes in Patients With Postacute Sequelae of COVID-19 Using Smartwatch Data: An Artificial Neural Network Approach. Arch. Phys. Med. Rehabil..

[14] Pennebaker J.W., Boyd R.L., Jordan K., Blackburn K. (2015). The Development and Psychometric Properties of LIWC2015.

[15] Nasreddine Z.S., Phillips N.A., Bédirian V., Charbonneau S., Whitehead V., Collin I., Cummings J.L., Chertkow H. (2005). The Montreal Cognitive Assessment, MoCA: A Brief Screening Tool For Mild Cognitive Impairment. J. Am. Geriatr. Soc..

[16] Arnett J.A., Labovitz S.S. (1995). Effect of physical layout in performance of the Trail Making Test. Psychol. Assess..

[17] Jonker C., Geerlings M.I., Schmand B. (2000). Are memory complaints predictive for dementia? A review of clinical and population-based studies. Int. J. Geriatr. Psychiatry.

[18] Watson D., Clark L.A., Tellegen A. (1988). Development and validation of brief measures of positive and negative affect: The PANAS scales. J. Personal. Soc. Psychol..

[19] Cohen S., Kamarck T., Mermelstein R. (1983). A Global Measure of Perceived Stress. J. Health Soc. Behav..

[20] Julian L.J. (2011). Measures of anxiety: State-Trait Anxiety Inventory (STAI), Beck Anxiety Inventory (BAI), and Hospital Anxiety and Depression Scale-Anxiety (HADS-A). Arthritis Care Res..

[21] Soto C.J., John O.P. (2017). The next Big Five Inventory (BFI-2): Developing and assessing a hierarchical model with 15 facets to enhance bandwidth, fidelity, and predictive power. J. Personal. Soc. Psychol..

[22] Rammstedt B., John O.P. (2007). Measuring personality in one minute or less: A 10-item short version of the Big Five Inventory in English and German. J. Res. Personal..

[23] Buysse D.J., Reynolds C.F., Monk T.H., Berman S.R., Kupfer D.J. (1989). The Pittsburgh sleep quality index: A new instrument for psychiatric practice and research. Psychiatry Res..

[24] Yesavage J.A., Sheikh J.I. (1986). 9/Geriatric Depression Scale (GDS). Clin. Gerontol..

[25] Cauce A.M., Felner R.D., Primavera J. (1982). Social support in high-risk adolescents: Structural components and adaptive impact. Am. J. Community Psychol..

[26] Nogueira I.B.R., Dias R.O.M., Rebello C.M., Costa E.A., Santana V.V., Rodrigues A.E., Ferreira A., Ribeiro A.M. (2022). A novel nested loop optimization problem based on deep neural networks and feasible operation regions definition for simultaneous material screening and process optimization. Chem. Eng. Res. Des..

[27] Bishop C. (2006). Pattern Recognition and Machine Learning.

[28] Powers D.M.W. (2011). Evaluation: From precision, recall and F-measure to ROC, informedness, markedness and correlation. arXiv.

[29] Julayanont P., Nasreddine Z.S., Larner A.J. (2017). Montreal Cognitive Assessment (MoCA): Concept and Clinical Review. Cognitive Screening Instruments: A Practical Approach.

[30] Dupuis K., Pichora-Fuller M.K., Chasteen A.L., Marchuk V., Singh G., Smith S.L. (2015). Effects of hearing and vision impairments on the Montreal Cognitive Assessment. Aging Neuropsychol. Cogn..

[31] Contemori G., Saccani M.S., Bonato M. (2024). Cognitive-Cognitive Dual-Task in Aging: A Cross-Sectional Online Study. PLoS ONE.

[32] Reid L.M., Maclullich A.M. (2006). Subjective memory complaints and cognitive impairment in older people. Dement. Geriatr. Cogn. Disord..

[33] Schöne C., Tandler S.S., Stiensmeier-Pelster J. (2015). Contingent self-esteem and vulnerability to depression: Academic contingent self-esteem predicts depressive symptoms in students. Front. Psychol..

[34] Kumar M., Srivastava S., Muhammad T. (2022). Relationship between physical activity and cognitive functioning among older Indian adults. Sci. Rep..

